# Abnormal downregulation of 10‐formyltetrahydrofolate dehydrogenase promotes the progression of oral squamous cell carcinoma by activating PI3K/Akt/Rb pathway

**DOI:** 10.1002/cam4.5327

**Published:** 2022-11-06

**Authors:** Yi Qu, Ying He, Hanjin Ruan, Lizheng Qin, Zhengxue Han

**Affiliations:** ^1^ Department of Oral and Maxillofacial & Head and Neck Oncology, Beijing Stomatological Hospital Capital Medical University Beijing China

**Keywords:** ALDH1L1, folic acid, oral squamous cell carcinoma, PI3K/Akt/Rb pathway, prognostic marker

## Abstract

**Background:**

10‐formyltetrahydrofolate dehydrogenase (ALDH1L1) is a major folate enzyme, which is usually underexpressed in malignant tumors and competes with tumors for the same folate substrate. However, the specific role and mechanisms of ALDH1L1 in oral squamous cell carcinoma (OSCC) remainsobscure.

**Methods:**

The expression level of ALDH1L1 in paired OSCC tissues and adjacent noncancerous tissues were detected by quantitative realtime PCR, Western blot and immunohistochemistry. The relationship between ALDH1L1 expression and clinical characteristics was analyzed. Besides, CCK8, EdU staining, colony formation, wound healing, transwell invasion, apoptosis, cell cycle assays and nude mice tumor bearing experiments were employed to assess the role of ALDH1L1 in OSCC. To explore the underlying mechanisms of these effects, cell cycle‐related markers were examined.

**Results:**

In this study, we revealed that ALDH1L1 expression was significantly reduced in OSCC, and its downregulation was associated with the malignancy of the tumor and poor prognosis of patients. In vivo and in vitro experiments, downregulation of ALDH1L1 in OSCC significantly inhibited the occurrence of NADP^+^‐dependent catalytic reactions and facilitated tumor cell growth, migration, invasion, survival, cell cycle progression, and xenograft tumor growth. On the contrary, re‐expression of ALDH1L1 plays a similar role to anti‐folate therapy, promoting NADPH production and suppressing the progression of OSCC. Furthermore, ALDH1L1 overexpressing obviously inhibited the expression of PI3K, p‐Akt, CDK2, CDK6, Cyclin D1, Cyclin D3, and Rb in OSCC cells, and promoted the expression of p27. LY294002 and 740 Y‐P were used to confirm the inhibitory effects of ALDH1L1 on OSCC progression through PI3K/Akt/Rb pathway.

**Conclusion:**

Our findings highlight the clinical value of ALDH1L1 as a prognostic marker and the potential of a new target for anti‐folate therapy.

## Graphical abstract

Anti‐folate therapy has achieved remarkable results in the treatment of a variety of solid malignancies. In the present study, we revealed that the folate enzyme ALDH1L1 inhibits OSCC progression by deactivating the PI3K/Akt/Rb signaling pathway. This provides a potential new target for anti‐folate therapy.

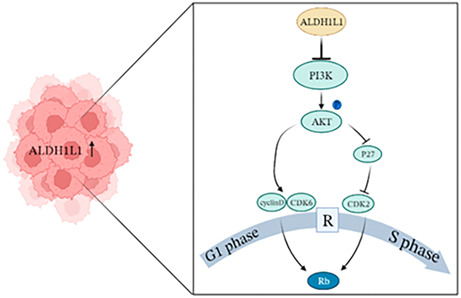

## 1 INTRODUCTION

Globally, head and neck cancers are very prevalent, have a high mortality rate, with over 500,000 new morbidities every year.^1^, ^2^ About 95% of these cancers are oral squamous cell carcinoma (OSCC), characterized by a high aggressiveness, repeated metastases as well as recurrence.^3^ The 5‐year survival rate for OSCC patients is about 50%.^4^ Folic acid (FA) is an important coenzyme in many one‐carbon unit transfer biochemical reactions, and is involved in the biosynthesis of several common amino acids, purine nucleotides, and thymic acid. These key biological pathways are essential for cell proliferation. Therefore, whether cancer patients can take FA supplements has been controversial in recent years.^5^ Abnormal FA metabolism is closely related to dysregulation of various folate metabolic enzymes in tumor cells.^6^ Therefore, understanding the role and molecular mechanism of abnormal FA metabolism enzymes in tumors is helpful to guide FA supplementation in OSCC patients and to provide potential prognostic markers and therapeutic targets.

10‐formyltetrahydrofolate dehydrogenase (ALDH1L1; also referred to as FDH), one of the most abundant folate enzymes in the cytoplasm, transforms 10‐formyl‐tetrahydrofolate (THF) into THF and CO_2_ through a NADP^+^‐mediated reaction, which involves the removal of one‐carbon groups in cells, thereby restricting their flux towards folate‐associated biosynthetic processes.^7^ Among humans, the ALDH1L1 gene has 23 exons that span about 77 kb on the long arm of chromosome 3 (3q21.2). The subsequent protein is a tetramer composed of 902 aa identical subunits.^8^ In addition, ALDH1L1 regulates cell de novo purine biosynthesis,^9^ degradation of formate^10^ as well as methylation status.^11^ These pathways are essential for cell proliferation as well as abnormal folate metabolism, and are associated with cancer.^12^

Recently, a study used The Cancer Genome Atlas (TCGA) data to evaluate gene expression profiles of 33 human cancer types. The results showed that compared with normal tissues, expressions of the ALDH1L1 gene are suppressed in early cancer stages, and are strongly inhibited in late cancer stages.^13^ In addition, to high extent, the ALDH1L1 gene is suppressed in high‐grade malignant tumors, when compared to low‐grade tumors. The consequences of up as well as downregulation of cell‐specific genes can be complicated, and may not be obvious.^14^ Compared with an alteration of gene expression, gene silencing is a much rare event in cancer, and it may be advantageous for the proliferative phenotype. Re‐expression of ALDH1L1 in tumor cells has been shown to lead to drastic anti‐proliferative outcomes, such as cell cycle arrest as well as apoptosis. Besides, intracellular accumulation of ALDH1L1 is associated with activation of p53 as well as its downstream target protein p21.^9^, ^15^, ^16^ Based on the existing researches, ALDH1L1 is expected to be the next target of anti‐folate therapy. However, the expression profile and function of ALDH1L1 are tumor‐specific. So far, the gene expression profiles, biological function, and regulation mechanism of ALDH1L1 in OSCC have not been established.

We found that the expression level of ALDH1L1 was significantly reduced in OSCC, and the downregulation of ALDH1L1 expression in tumor tissues was associated with poor prognosis of patients. Furthermore, downregulation of ALDH1L1 in OSCC is conducive to limit the occurrence of NADP^+^‐dependent reactions to meet the needs of rapid development of tumor. When ALDH1L1 is overexpressed in OSCC as a therapeutic target, it deactivates the PI3K/Akt/Rb pathway and inhibits the rapid progression of OSCC. Therefore, ALDH1L1 may serve as a potential prognostic marker for OSCC and a new therapeutic target for anti‐folate therapy.

## 2 MATERIALS AND METHODS

### 2.1 Tissue samples and clinicopathological data

A cohort study was designed which included 67 patients who were diagnosed as primary OSCC by histopathology and admitted at Beijing Stomatology Hospital, Capital Medical University from January 2010 to January 2015. All patients had not received treatment before and had no history of radiotherapy or chemotherapy. The baseline information and clinical outcomes were collected and updated every 3 months. The tumor staging and grading were achieved according to the AJCC and WHO guidelines. Lymphatic invasion (LI) or vascular invasion (VI) was diagnosed if aggregates tumor cells in endothelial‐lined channels of lymphatic or blood vessels were present. The follow‐up was carried out from the surgical date to the death or final follow‐up date. OSCC samples and those of adjacent normal tissues (ANTs) obtained after surgical resection were immediately divided into two parts. One part was immediately frozen and kept at −80°C for western blot and real‐time quantitative reverse transcription‐PCR (qRT‐PCR) analyses, while the other part for immunohistochemistry (IHC) staining was fixed with 4% paraformaldehyde and paraffin‐embedded before processing. All the above‐mentioned information and samples were collected in accordance with the Declaration of Helsinki. Approvals were obtained from every patient and from the Research Ethics Committee of Beijing Stomatology Hospital for research purposes.

### 2.2 RNA extraction and qRT‐PCR

Total RNA extraction was done by the TRIzol reagent (CWbiotech, China). RNA concentration and purity were assessed followed by cDNA synthesis using the Prime Script RT Reagent Kit (Takara, Japan). qRT‐PCR was carried out utilizing SYBR Green PCR kit (Qiagen, Germany). Relative mRNA expressions were compared by the 2^−ΔΔCt^ method after normalization to GAPDH expression. The primers were designed and provided by Life Technologies and the sequence is listed in Table S1.

### 2.3 Protein extraction and Western blotting

Extraction of total proteins was done using the Tissue Protein Extraction Kit (CWbiotech, China). The lysates and immunoblotting were performed as previously described.^17^ The primary as well as secondary antibodies are described in Table S2.

### 2.4 Immunohistochemistry

Tissue samples were fixed in paraformaldehyde (4%), dehydrated, and soaked in paraffin. Paraffin‐embedded tissues collected above were employed for IHC. IHC staining and score of staining were performed as previously described.^17^ The primary as well as secondary antibodies are described in Table S2.

### 2.5 Animal experiments

Approvals for animal experiments were obtained from the Institutional Research Ethics Committee for animal of the Stomatology Hospital of Capital Medical University. Experimental procedures were in accordance with the national guidelines for the care and use of laboratory animals. Female BALB/c nude mice (4 weeks old) were obtained from the Beijing HFK Bioscience (China) and randomly divided into each group. For tumor growth analysis, the pooled cultures of sub‐cell lines infected with lentiviruses and selected with puromycin (i.e., CAL‐27‐sh‐NC, CAL‐27‐sh‐ALDH1L1, SCC‐25‐LV‐Ctrl, SCC‐25‐LV‐ALDH1L1) were subcutaneously transplanted into mice left or right flanks (1 × 10^6^ CAL‐27 or 2.5 × 10^6^ SCC‐25 cells per mouse). After tumor formation, their lengths (L) and widths (W) were determined using digital Vernier calipers, every 3 days. Then, tumor volumes were determined by: Tumor volume = W^2^ × L/2. A month after tumor formation, mice were anestheticized and killed. The primary tumors were resected, fixed, and embedded in paraffin for IHC analyses.

### 2.6 Cell culture and reagents

TIGKs, SCC‐4, CAL‐27, SCC‐25, and SCC‐9 cells were procured from the American Type Culture Collection (ATCC). HOK cells were obtained from Shanxi Medical University School and Hospital of Stomatology. TIGKs cells were cultured in Dermal Cell Basal Medium (PCS‐200‐030, ATCC) supplemented with Keratinocyte Growth Kit (PCS‐200‐040, ATCC). CAL‐27 cells were appropriately cultured in DMEM (SH30243.01, Hyclone) with 10% FBS as well as 0.5% penicillin/streptavidin. SCC‐4, SCC‐25, and SCC‐9 cells were seeded in DMEM/F‐12 medium (11330032, Gibco) with 10% FBS, 0.5% penicillin‐streptavidin mixture and 400 ng/mL hydrocortisone. HOK cells were seeded in Keratinocyte SFM medium (10744019, Thermo Fisher Scientific). Cells were kept in a humidified incubator at 37°C with 5% CO_2_. LY294002 (PI3K inhibitor) and 740 Y‐P (PI3K activator) were ordered from MCE (Shanghai, China).

### 2.7 Plasmids and lentiviruses

Knockdown lentivirus expressing negative control shRNA (sh‐NC) and shRNA targeting ALDH1L1 (sh‐ALDH1L1) were constructed using the pGC‐FU‐3FLAG‐SV40‐EGFP‐IRES‐puromycin vector followed by packaging in 293 T cells (puromycin resistance). The overexpression lentivirus expressing ALDH1L1 (LV‐ALDH1L1) and control lentivirus vectors (LV‐Ctrl) were established and packaged by GeneChem (Shanghai, China; puromycin resistance). Tables S3 and S4 show their sequences. For lentivirus infection, different OSCC cell lines were transfected with lentiviral vectors (an MOI of about 10) using polybrene (6 μg/mL) for 16 h. At 48 h post‐infection, 2.5 μg/mL puromycin (Invitrogen, USA) was used for stable cell pool selecting. Stable overexpression and knockdown cell lines were established as described previously.^18^

### 2.8 Evaluation of cell growth, proliferation, and survival

Cell growth was assessed by the Cell counting kit‐8 (CCK8) assay. The assay was performed according to the manufacturer's instructions.^17^ Labelling of proliferating cells was done by 5‐ethynyl‐2′‐deoxyuridine (EdU) staining, as previously reported.^19^ Anchorage‐dependent growth abilities (cell survival) were evaluated by the colony formation assay. In brief, cell seeding was done in 6‐well plates at 5000 cells/per well. After 10 days, cells were subjected to formaldehyde fixation, crystal violet staining, photographing, and counting.

### 2.9 Cellular migration as well as invasion assays

The wound healing assay was performed to assess migration abilities as previously described.^17^ The BioCoat Matrigel invasion chambers (BD Biosciences) were used for the invasion assays. In brief, suspension of 1 × 10^5^ cells in serum‐free medium was placed in the top chamber. Then, 10% FBS‐supplemented medium was added to the lower chamber, followed by 24 or 48 h of incubation. Cells that had migrated to the lower membrane surface were stained, imaged, and enumerated in six random fields in each group by microscopy. DMSO (vehicle), LY294002, or 740 Y‐P were added to the top as well as bottom of transwell chambers. Experiments were conducted in triplicates.

### 2.10 Cell apoptosis assay

OSCC cell line abundances in early as well as late apoptosis were assessed by AnnexinV‐7‐ Amino‐Actinomycin (7‐AAD)/Phycoerythrin (PE) staining (BD Biosciences, USA) according to the instructions of the manufacturer. Definition of early and late apoptosis was done by 7‐AAD^−−^/PE^+^ and 7‐AAD^+^/PE^+^ staining.

### 2.11 Cell cycle analysis

Harvested cells were washed and fixed in cold ethanol (70%) for 2 h at −20°C. Then, cells were washed twice using PBS and once in a BD Pharmingen staining buffer (BD Biosciences, 554,656). Resuspension of cell pellets was done in BD Pharmingen PI/RNase staining buffer (0.5 ml; BD Biosciences, 550,825) followed by 15 min of incubation at room temperature (RT). Detection of red fluorescence was done at an excitation wavelength of 488 nm, after which the abundance of cells at G0/G1, S, as well as G2/M phases were determined by the ModFit software.

### 2.12 NADPH/NADP ^+^ ratio detection

The NADPH/NADP^+^ ratio was determined by using NADP^+^/NADPH.

Assay Kit with WST‐8 (S0179, Beyotime), as previously reported.^20^

### 2.13 Statistical analysis

Data are expressed as mean ± SD for at least *n* = 3. Statistical analyses were done by SPSS V19.0 software. Comparisons of means between and among groups were done by t‐test and one‐way ANOVA, respectively. Student's *t*‐test, χ^2^ test, and Fisher's exact test were used to compare correlations between ALDH1L1 expression levels and clinic‐pathologic characteristics. Survival assessments were done by the Kaplan–Meier method, while comparisons of curves were done by the log‐rank test. The Cox proportional hazards model was used to investigate the prognostic factors by univariate analysis while the effects of confounding factors were adjusted for multivariate analysis. *p* < 0.05 was considered significant.

## 3 RESULTS

### 3.1 ALDH1L1 is decreased in OSCC patients and cell lines, and its lower expression predicts worse prognosis

**FIGURE 1:**
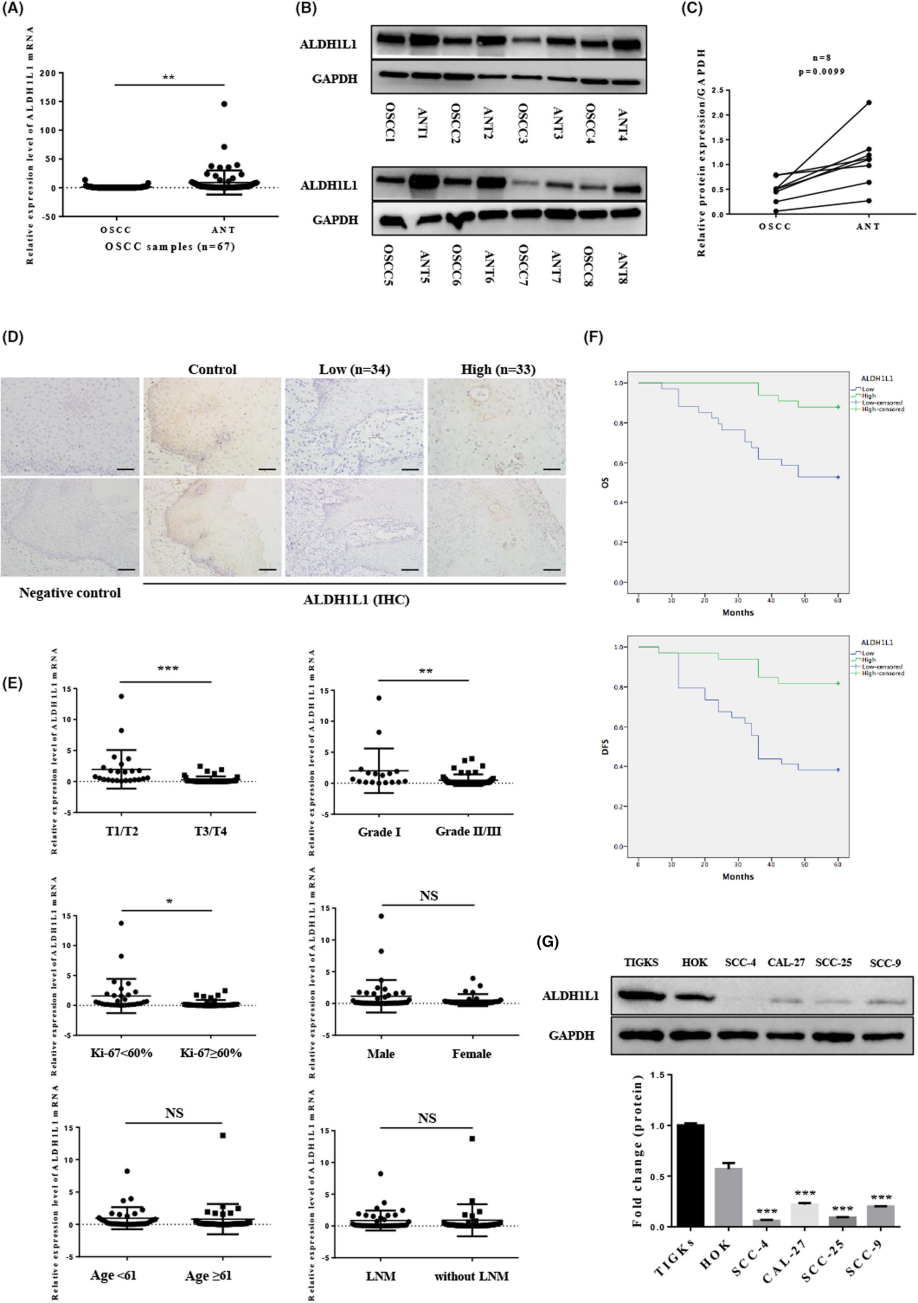
ALDH1L1 expression in OSCC tissues and cell lines. (A) qRT‐PCR analysis of ALDH1L1 expression in 67 pairs of OSCC and cancer‐adjacent normal tissues (ANTs). (B, C) Representative data of ALDH1L1 protein expression in eight paired OSCC tissues and ANTs were detected via western blot analysis. (D) IHC staining of ALDH1L1 in control (ANTs) and OSCC tissues. The negative control was established by using PBS as a substitute for the primary antibody. Scale bar: 20 μm (top) and 50 μm (bottom). (E) The correlation between ALDH1L1 mRNA expression levels and clinical features in OSCC patients. (F) Kaplan–Meier survival analysis was performed to estimate the relationship between ALDH1L1 protein expression in tumor tissues and the overall survival (OS) (*p* = 0.0002) and disease‐free survival (DFS) (*p* = 0.001) of OSCC patients. (G) ALDH1L1 protein expression in TIGKS, HOK and OSCC cell lines, including SCC‐4, CAL‐27, SCC‐25, and SCC‐9. Error bars show mean ± SD. *p < 0.05, **p < 0.01, ***p < 0.001.

To understand the involvement of ALDH1L1 in OSCC, we detected ALDH1L1 mRNA and protein expression of 67 paired OSCC and ANTs by qRT‐PCR assay and Western blot analysis. mRNA levels of ALDH1L1 were markedly suppressed in OSCC tissues, compared to ANTs (*p* = 0.0021; Figure 1A). In addition, expressions of ALDH1L1 protein were in accordance with mRNA findings (Figure 1B, C). Moreover, IHC staining revealed that ALDH1L1 was localized in the cytoplasm, and was decreased or even silenced in all the OSCC tissues, while most ANTs showed strong positive. The representative examples of staining are shown in Figure 1D.

Then, we analyzed whether the low expression of ALDH1L1 is associated with clinic‐pathological parameters of OSCC patients. The relationship between ALDH1L1 mRNA and various clinicopathological features is shown in Figure 1 E, and Table 1. According to the results, suppressed ALDH1L1 levels were clearly correlated with clinical T3/T4 stages (*p* = 0.0009), advanced pathological grade (*p* = 0.008) and high positive rate of Ki‐67 (*p* = 0.0108). Similarly, associations between ALDH1L1 protein levels and clinic‐pathological characteristics of OSCC were assessed (Table 2). In high‐ and low‐ALDH1L1 expression groups, clinical T stage (*p* = 0.0001), pathological grade (*p* = 0.0001), and Ki‐67 status (*p* = 0.002) were markedly different. Kaplan–Meier analysis showed that, compared to high expressions, low ALDH1L1 expression was associated with markedly decreased overall survival (OS) (*p* = 0.0002) and disease‐free survival (DFS) outcomes (*p* = 0.001) (Figure 1F). The OS and DFS rates were 55% and 40% for the low‐ALDH1L1 group, respectively, compared with 90% and 80% for the high‐ALDH1L1 group. Univariate analysis showed that clinical lymph node metastasis, T stage, Ki‐67, and ALDH1L1 were correlated with OS in patients with OSCC (Table 3). In addition, in multivariate analysis, only Ki‐67 index was significant independent predictor of OS in OSCC patients (Table 3).

**TABLE 1:** Relationship between ALDH1L1 mRNA expression and the clinicopathological characteristics of the 67 OSCC patients

Variables	All cases		ALDH1L1 ^2−△△CT^ ^b^	*p*‐value
	No.	%	Mean ± SD	
Age^a^				0.7671
<61	31	46.3	0.96 ± 0.30	
≥61	36	53.7	0.81 ± 0.39	
Gender				0.2332
Male	39	58.2	1.13 ± 0.41	
Female	28	41.8	0.52 ± 0.18	
Clinical T stage				0.0009***
T1/T2	24	35.8	1.97 ± 0.63	
T3/T4	43	64.2	0.27 ± 0.08	
Pathological grade				0.0075**
I	17	25.4	2.01 ± 0.87	
II/III	50	74.6	0.49 ± 0.13	
Lymph node metastasis				0.933
Absent	31	46.3	0.90 ± 0.45	
Present	36	53.7	0.86 ± 0.26	
Ki‐67^c^				0.0108*
<60%	30	44.8	1.58 ± 0.52	
≥60%	37	55.2	0.31 ± 0.10	
Lymphatic invasion				0.9667
Absent	62	92.5	0.88 ± 2.08	
Present	5	7.5	0.92 ± 1.40	
Vascular invasion				0.4969
Absent	64	95.5	0.92 ± 2.08	
Present	3	4.5	0.08 ± 0.07	

Abbreviations: SD, standard deviation.

* *p* < 0.05.

** *p* < 0.01.

*** *p* < 0.001.

^a^ 61 years is the median age of the subjects.

^b^ 2^−△△CT^ indicates the difference in the cycle number at which a sample's fluorescent signal passes a given threshold above baseline (Ct) derived from a specific gene compared with that of GAPDH in tumor tissues.

^c^ Percentage of Ki‐67 positive cells, 60% is the median value of the subjects.

**TABLE 2:** Relationship between ALDH1L1 protein expression and clinicopathological characteristics of the 67 OSCC patients.

Variables	All cases	ALDH1L1		*p*‐value^b^
		Low	High	
Age^a^				0.396
<61	31	14	17	
≥61	36	20	16	
Gender				0.918
Male	39	20	19	
Female	28	14	14	
Clinical T stage				0.0001***
T1/T2	24	1	23	
T3/T4	43	33	10	
Pathological grade				0.001**
I	17	2	15	
II/III	50	32	18	
Lymph node metastasis				0.396
Absent	31	14	17	
Present	36	20	16	
Ki‐67^c^				0.002**
<60%	30	9	21	
≥60%	37	25	12	
Lymphatic invasion				0.673
Absent	62	32	30	
Present	5	2	3	
Vascular invasion				1.000
Absent	64	32	32	
Present	3	2	1	

** *p* < 0.01.

*** *p* < 0.001.

^a^ 61 years is the median age of the subjects.

^b^ Chi‐square test or Fisher' s exact test.

^c^ Percentage of Ki‐67 positive cells, 60% is the median value of the subjects.

**TABLE 3:** Univariate and multivariate analyses on survival in OSCC patients

Characteristics	Subset	Hazard ratio (95% CI)	*p*‐value
Univariate analysis			
Gender	Female/Male	1.745 (0.670‐4.544)	0.254
Age	Age<61/Age≥61	0.835 (0.348‐2.007)	0.687
Clinical T stage	T1, T2/T3,T4	6.194 (1.436‐26.718)	0.014*
Pathological grade	I/II, III	3.613 (0.838‐15.578)	0.085
Lymph node metastasis	Absent/present	4.505 (1.502‐13.514)	0.007**
Ki‐67	<60%/≥61%	73.124 (1.973‐2709.592)	0.02*
ALDH1L1	Low/High	0.196 (0.065‐0.586)	0.004**
Lymphatic invasion	Absent/present	2.813(0.824‐9.607)	0.099
Vascular invasion	Absent/present	2.603(0.603‐11.230)	0.200
Multivariate			
Ki‐67	<60%/≥61%	73.124 (1.973‐2709.592)	0.02*

* *p* < 0.05.

** *p* < 0.01.

Subsequently, we tested four human OSCC cell lines (SCC‐25, CAL‐27, SCC‐4, SCC‐9) for ALDH1L1 expression. Human OSCC cell lines were found to have significantly lower ALDH1L1 protein levels, relative to the human immortal gingival epithelial cell line TIGKs and human oral mucosa keratinocyte HOK, respectively (Figure 1G). In addition, CAL‐27 and SCC‐9 exhibited elevated ALDH1L1 levels compared to SCC‐4 and SCC‐25.

### 3.2 Downregulation of ALDH1L1 promoted cell growth and reduced the proportion of NADPH/NADP ^+^ in vitro

**FIGURE 2:**
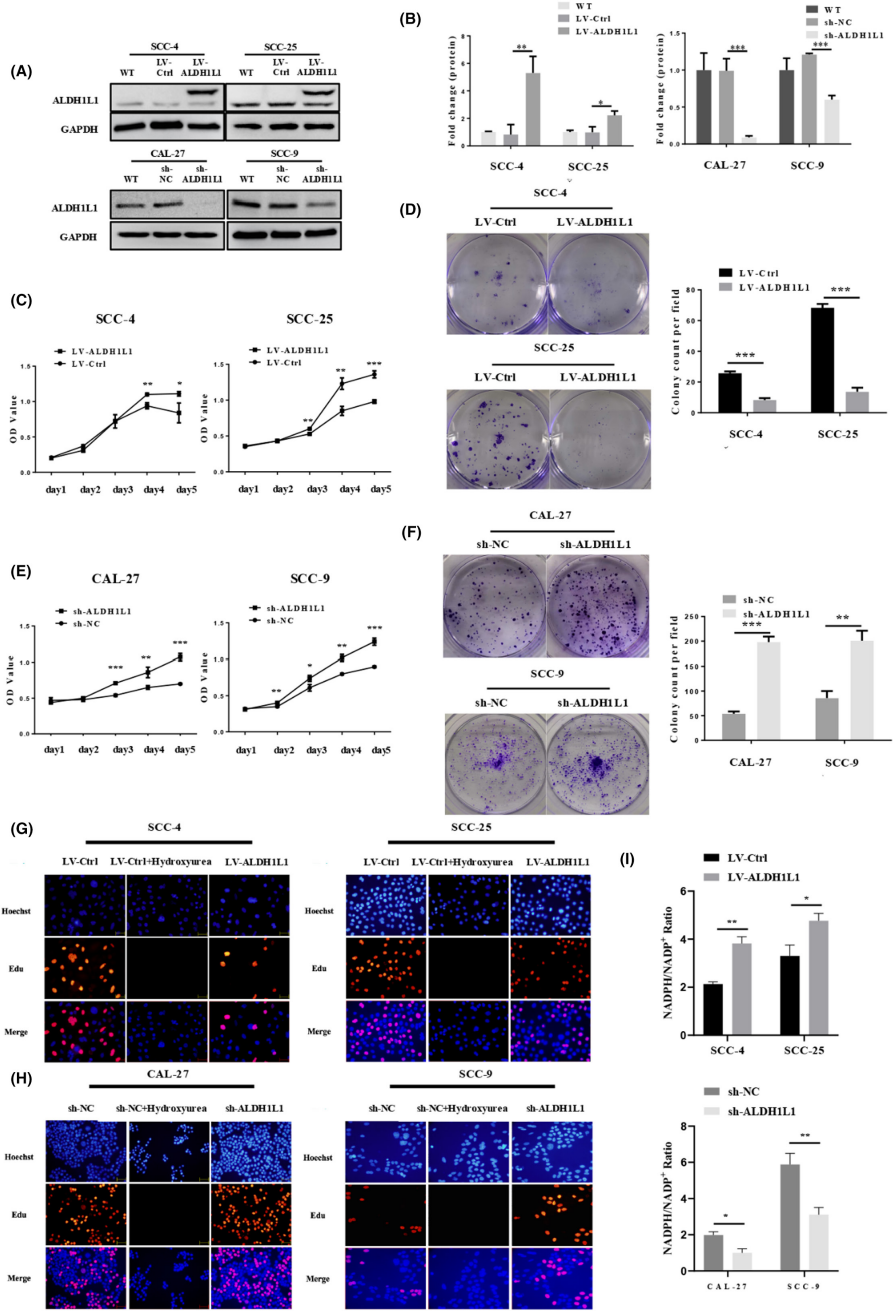
Downregulation of ALDH1L1 facilitates the growth, proliferation, and colony‐forming ability of OSCC cells. (A, B) Western blot analysis of ALDH1L1 overexpression and downregulation of lentivirus transfected SCC‐4, SCC‐25, and CAL‐27, SCC‐9 compared with the control lentivirus. (C, D) Overexpression of ALDH1L1 suppressed SCC‐4 and SCC‐25 cell growth and clonogenic ability in vitro by using CCK8 and clonogenic assays. (E, F) ALDH1L1 knockdown enhanced CAL‐27 and SCC‐9 cell growth and colonogenic ability in vitro. (G, H) OSCC cells were subjected to EdU staining. Typical images of EdU‐stained proliferating cell nuclei (red) and Hoechst‐stained cell nuclei (blue) and merged images are shown. Hydroxyurea is a DNA synthesis inhibitor (10 mM, 0.5 h). (I) ALDH1L1 downregulation remarkably reduced the proportion of NADPH/NADP+, whereas ALDH1L1 overexpression increased the proportion of NADPH/NADP+ in OSCC cells. Data were confirmed in duplicate trials. Error bars show mean ± SD. *p < 0.05, **p < 0.01, ***p < 0.001.

Prompted by the above findings, we examined the function of ALDH1L1 in OSCC. We stably knockdown ALDH1L1 in CAL‐27 and SCC‐9 cells. Lentiviral infection was used to overexpress ALDH1L1 in SCC‐4 and SCC‐25 cells. These experiments were verified by western blot (Figure 2A, B). The ALDH1L1 overexpression efficiency in LV‐ALDH1L1 SCC‐4 and SCC‐25 cells were 6.39 and 2.23‐fold, and the ALDH1L1 knockdown efficiency in sh‐ALDH1L1 CAL‐27 and SCC‐9 cells were 91.3% and 51%. According to the results of CCK8, EdU, and clone formation assays, we found that downregulation of target gene in the relatively high expression of ALDH1L1 cell lines CAL‐27 and SCC‐9 significantly enhanced cell growth, proliferation as well as clonogenic abilities (Figure 2 E, F, H, and Figure S1A). However, upregulation of ALDH1L1 in SCC‐4 and SCC‐25 cell lines with relatively low ALDH1L1 expression obviously inhibited cell growth, proliferation as well as colony formation (Figure 2C, D, G, and Figure S1B). In addition, downregulation of ALDH1L1 in OSCC cells could also inhibit NADP^+^‐dependent responses and reduce the production of NADPH. On the contrary, upregulation of ALDH1L1 promoted the NADPH/NADP^+^ ratio in SCC‐4 and SCC‐25 cells (Figure 2I). In summary, ALDH1L1 expression alters OSCC cell growth, proliferation, cloning as well as NADP^+^‐dependent biological responses.

### 3.3 ALDH1L1 re‐expression suppresses cell migration, invasion, and promotes cell cycle arrest in vitro

**FIGURE 3:**
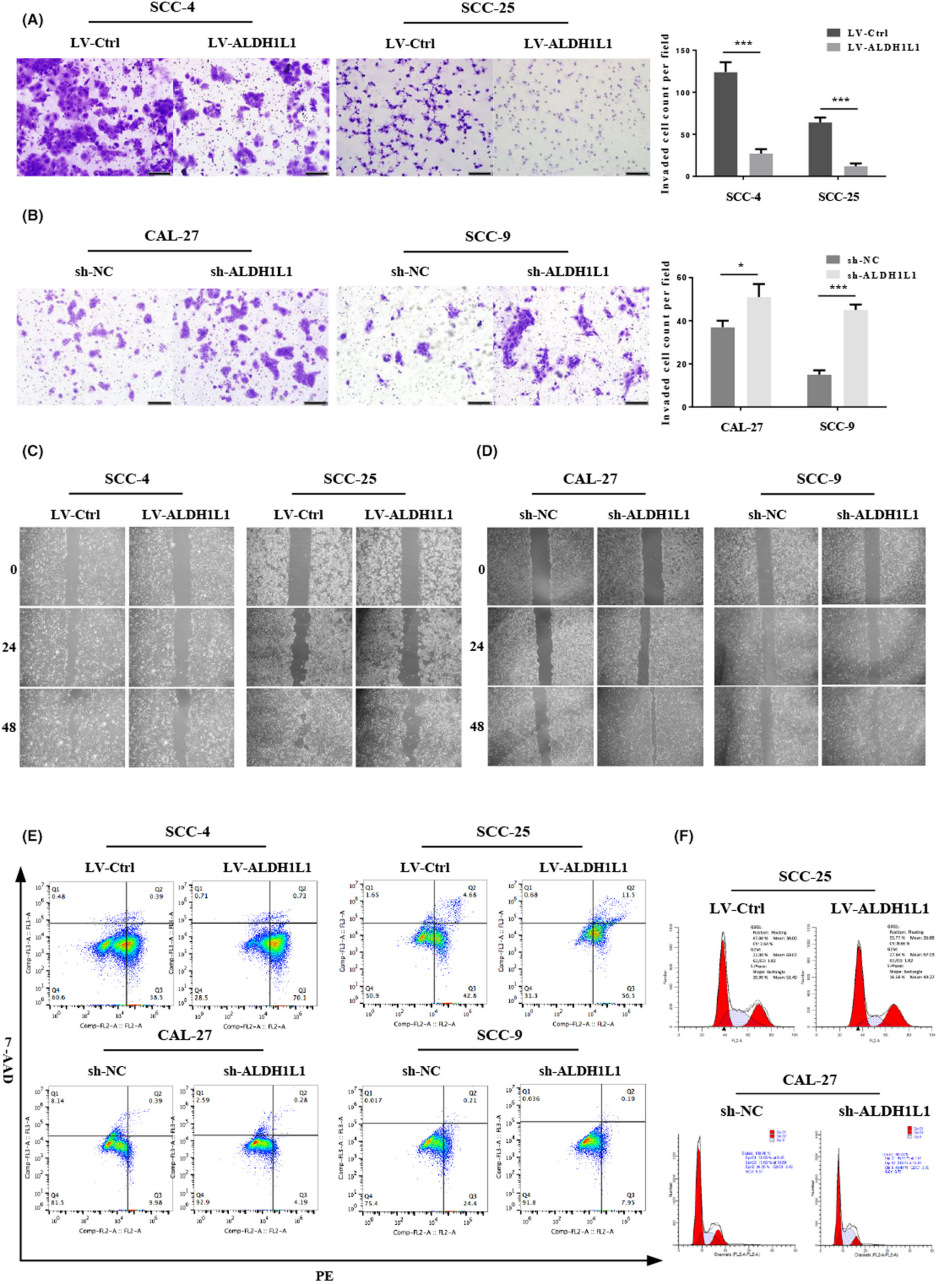
Downregulation of ALDH1L1 promoted cell migration, invasion, cell cycle arrest, and suppressed cell apoptosis in vitro. (A, C) Upregulation of ALDH1L1 inhibited SCC‐4 and SCC‐25 cells’ invasion and migration. Scale bar: 200 μm. (B, D) Downregulation of ALDH1L1 enhanced CAL‐27 and SCC‐9 cell invasion and migration. Scale bar: 200 μm. (E) The cell apoptosis distribution after transfection was detected by flow cytometry. (F) Representative plots of the cell cycle phases are shown for the SCC‐25 and CAL‐27 cells. The representative images were from at least three independent experiments. *p < 0.05, ***p < 0.001.

We evaluated the effects of ALDH1L1 on OSCC cell migration, invasion as well as apoptosis. Downregulation of ALDH1L1 significantly accelerated cell migration and invasion as revealed by scratch‐wound (Figure 3D and Figure S2B) and transwell assays (Figure 3B), respectively. On the contrary, upregulation of ALDH1L1 significantly suppressed the migration (Figure 3C and Figure S2A) and invasion (Figure 3A) ability of SCC‐4 as well as SCC‐25 cells. Furthermore, annexinV‐7‐AAD/PE double staining assay of sh‐ALDH1L1‐infected OSCC cells markedly reduced the number of early‐ and late‐apoptotic cells, while that in LV‐ALDH1L1 SCC‐4 and SCC‐25 cells were significantly increased, confirming the apoptotic effects of ALDH1L1 on OSCC cells (Figure 3 E and Figure S2C).

We also observed changes in cell cycle following ALDH1L1 down or upregulation in OSCC cells. Flow Cytometry showed that knockdown of ALDH1L1 in CAL‐27 cells led to promotion of cell cycle progression with more cells in the S phases (Figure 3F and Figure S2D). In contrast, overexpression of ALDH1L1 in SCC‐25 cells led to arrests of the cell cycle in the G0/G1 and G2/M phases, which were accompanied by less cells in the S phase (Figure 3F and Figure S2D). hese results imply that ALDH1L1 is a potential suppressor of OSCC progression.

### 3.4 ALDH1L1 knockdown facilitates tumorigenesis in a xenograft model

**FIGURE 4:**
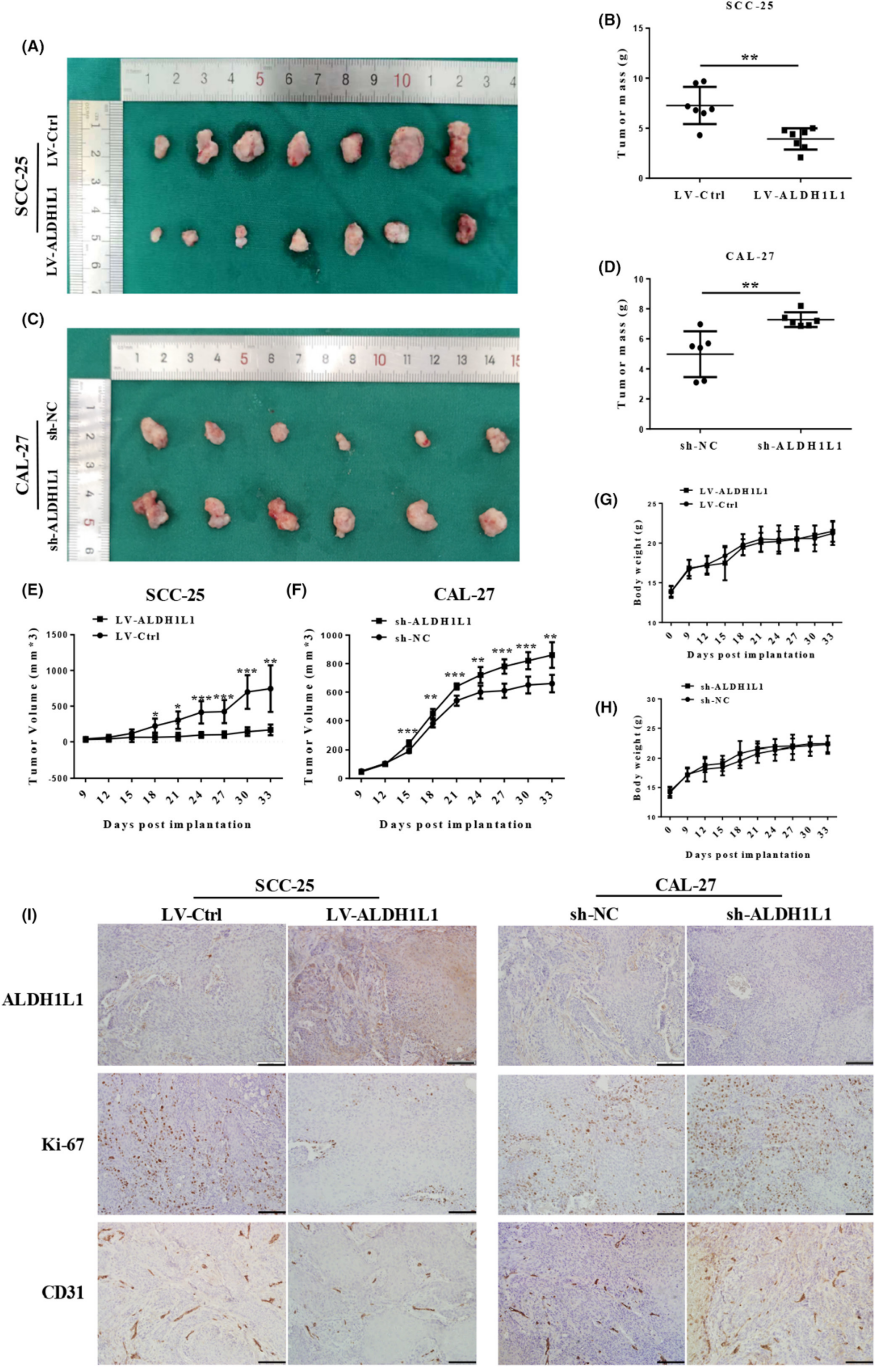
Downregulation of ALDH1L1 accelerates tumorigenesis in xenograft models. (A‐D) SCC‐25‐LV‐ALDH1L1, CAL‐27‐sh‐ALDH1L1, or their controls were injected subcutaneously into the back of BALB/c‐nu mice to compare the tumorigenic ability. These graphs show the tumor xenografts 1 month after ectopic‐subcutaneous implantation in nude mice. The tumor weight was observed. (E, F) The tumor volume was observed every 3 days after tumor formation. (G, H) The body weight of mice was observed every 3 days after tumor formation in four groups. (I) IHC staining of ALDH1L1, Ki‐67, and CD31 in the tumor samples of SCC‐25‐LV‐ALDH1L1, CAL‐27‐sh‐ALDH1L1, and their control groups. Scale bar: 100 μm. Error bars show mean ± SD. *p < 0.05, **p < 0.01, ***p < 0.001.

For in vivo experiments, OSCC stable sub‐cell lines (CAL‐27‐sh‐NC, CAL‐27‐sh‐ALDH1L1, SCC‐25‐LV‐Ctrl, SCC‐25‐LV‐ALDH1L1) were injected in nude mice. Xenografts of sh‐ALDH1L1 groups exhibited low levels of ALDH1L1, while those of the LV‐ALDH1L1 groups expressed high levels of ALDH1L1, relative to the control groups (Figure 4I and Figure S3A). Our results showed that tumor volumes as well as weights and tumor cell proliferation (as assessed by Ki‐67 index) were increased in the mice injected with ALDH1L1‐knockdown CAL‐27 cells compared with the control group (Figure 4C, D, F, and I, Figure S3B). Conversely, ALDH1L1‐overexpressing SCC‐25 cells resulted in formation of significantly small tumor volumes, weights, and suppressed tumor cell proliferation, relative to control group (Figure 4A, B, E, and I, Figure S3B). There were no marked differences in weight changes for mice in experimental and control groups (Figure 4G, H). Based on detection of CD31^+^ vascular structures, vessel densities were also verified to be significantly increased in ALDH1L1‐knockdown tumors and decreased in ALDH1L1‐overexpressing tumors, relative to control groups (Figure 4I and Figure S3C). These in vitro and in vivo results suggested that the downregulation of ALDH1L1 in OSCC is conducive to the demand for rapid growth of tumor cells, and the upregulation of ALDH1L1 as a target can significantly inhibit tumor development.

### 3.5 Upregulation of ALDH1L1 deactivates PI3K/Akt/Rb signaling pathway that regulates G1/S progression in OSCC

**FIGURE 5:**
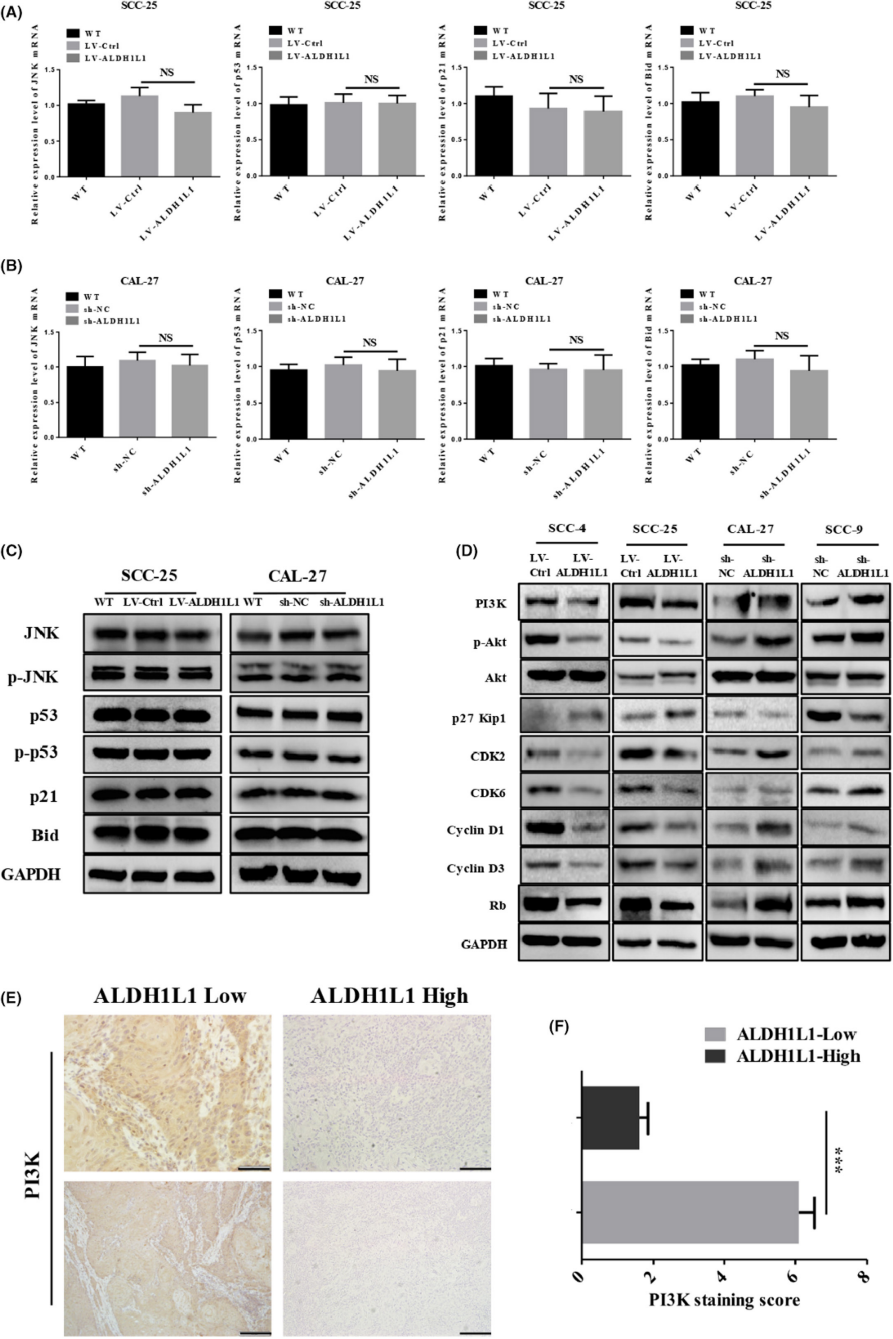
Upregulation of ALDH1L1 inhibited the activation of PI3K/Akt/Rb signaling pathway in OSCC cells. (A, B) The mRNA expression levels of JNK, p53, p21, and Bid in SCC‐25‐LV‐ALDH1L1, CAL‐27‐sh‐ALDH1L1, and their corresponding control cells were examined by qRT‐PCR analysis. (C) The expression levels of p53, p‐p53, p21, JNK, p‐JNK, and Bid protein in SCC‐25‐LV‐ALDH1L1, CAL‐27‐sh‐ALDH1L1, and their control cells were examined by western blot analysis. (D) Western blot was used to detect the expression changes of key regulatory proteins of PI3K/Akt/Rb pathway in the four groups of cells SCC‐25‐LV‐Ctrl, SCC‐25‐LV‐ALDH1L1, CAL‐27‐sh‐NC, and CAL‐27‐sh‐ALDH1L1. (E) Representative images of IHC staining for PI3K in ALDH1L1 expression low or high groups. Scale bar: 50 μm (top) and 100 μm (bottom). (F) Results of IHC staining were evaluated by the staining scores. Error bars show mean ± SD. *p < 0.05, **p < 0.01, ***p < 0.001.

ALDH1L1 protein levels are correlated with various regulatory proteins, such as p53, p21, JNK, and Bid in different tumor types, implying that it affects cell proliferation as well as survival through the associated signal pathways.^15^ Thus, we evaluated the activations of p53, p‐p53, p21, JNK, p‐JNK, and Bid in both ALDH1L1‐knockdown and ‐overexpressing OSCC cells. However, knocked down or overexpressed ALDH1L1 had no significant effect on p21, Bid, and the phosphorylation of p53 and JNK (Figure 5A–C, Figure S4A, B). According to the results of cell cycle assays and previous studies,^16^ the regulation of ALDH1L1 is associated with progression of the cell cycle, and the classic PI3K/Akt/Rb pathway plays a vital role in cell cycle progressions.^21^ Therefore, we simultaneously detected key regulatory proteins of this pathway in four groups of cells (CAL‐27‐sh‐NC, CAL‐27‐sh‐ALDH1L1, SCC‐25‐LV‐Ctrl, SCC‐25‐LV‐ALDH1L1). The results showed that PI3K, p‐Akt, CDK2, CDK6, Cyclin D1, Cyclin D3, and Rb were significantly increased in ALDH1L1‐knockdown cells, and p27 was significantly decreased. In contrast, ALDH1L1 overexpressing obviously inhibited the expression of PI3K, p‐Akt, CDK2, CDK6, Cyclin D1, Cyclin D3, and Rb in OSCC cells, and promoted the expression of p27 (Figure 5D, Figure S4C–F).

Subsequently, we used IHC to detect PI3K expressions in OSCC tissues, to verify its correlation with ALDH1L1. Consistent with in vitro results, ALDH1L1 expression was inversely correlated with PI3K protein expression in 40 clinical OSCC tissues. The low expression of ALDH1L1 was markedly correlated with high expression levels of PI3K protein (Figure 5 E, F). Kaplan–Meier analysis showed that, the ALDH1L1 high expression/PI3K low expression combination had a significantly better OS (ALDH1L1^high^/PI3K^low^ vs. ALDH1L1^low^/PI3K^low^ *p* = 0.041, ALDH1L1^high^/PI3K^low^ vs. ALDH1L1^low^/PI3K^high^ *p* = 0.001, ALDH1L1^high^/PI3K^low^ vs. ALDH1L1^high^/PI3K^high^ *p* = 0.005), and DFS (ALDH1L1^high^/PI3K^low^ vs. ALDH1L1^low^/PI3K^low^ *p* = 0.038, ALDH1L1^high^/PI3K^low^ vs. ALDH1L1^low^/PI3K^high^ *p* = 0.001, ALDH1L1^high^/PI3K^low^ vs. ALDH1L1^high^/PI3K^high^ *p* = 0.002) than the other three groups. Furthermore, patients with low ALDH1L1 expression/low PI3K expression or high ALDH1L1 expression/high PI3K expression had a better OS and DFS than those with low ALDH1L1 expression/high PI3K expression, although the difference was not statistically significant (Figure S5A, B).

### 3.6 740 Y‐P reversed the inhibitory effect of ALDH1L1 on OSCC

**FIGURE 6:**
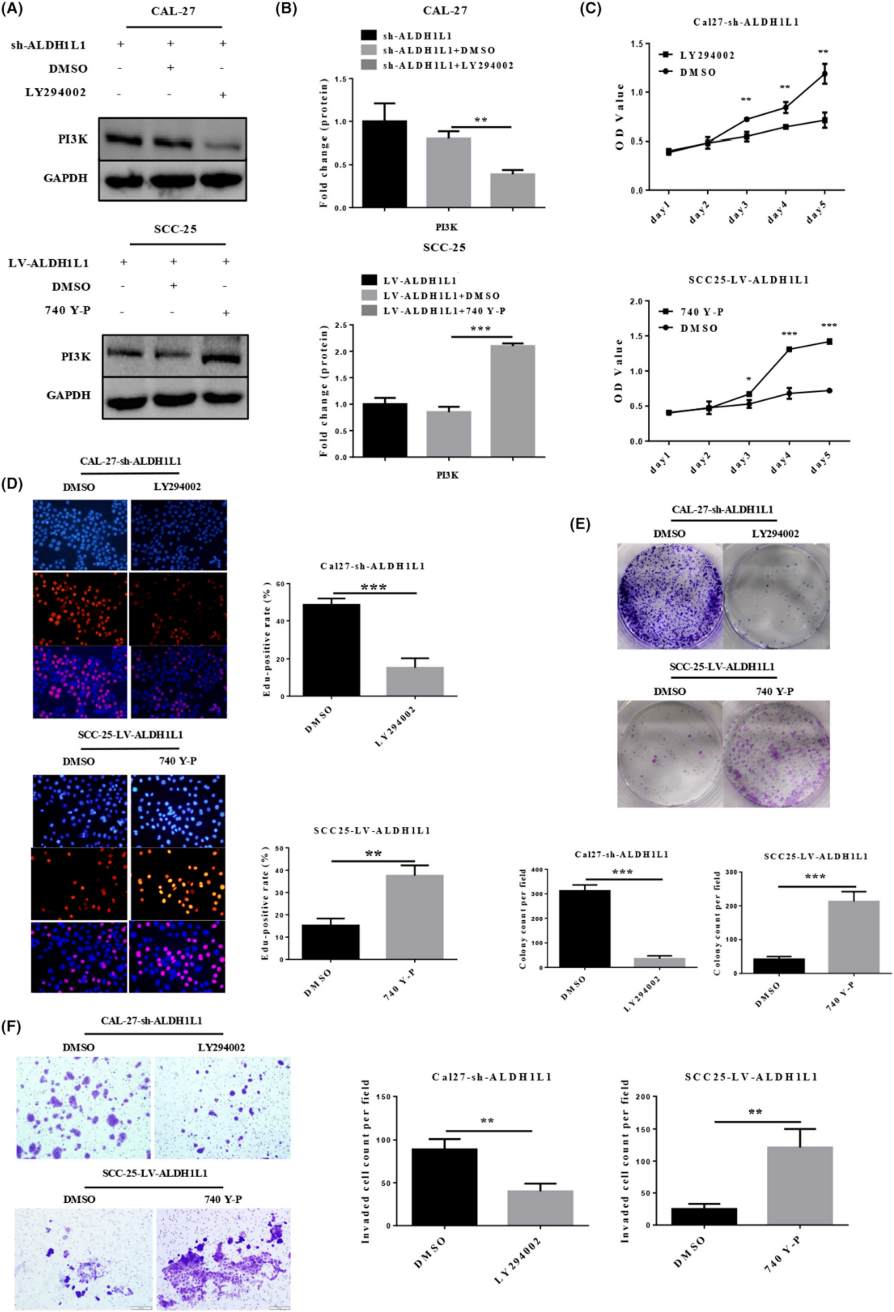
740 Y‐P rescued the inhibitory effects of ALDH1L1 on OSCC growth, proliferation, cloning, and invasion (A, B) Western blot analysis of PI3K protein expression in CAL‐27‐sh‐ALDH1L1 cells added PI3K inhibitor LY294002 and SCC‐25‐LV‐ALDH1L1 cells added PI3K activator 740 Y‐P. (C‐F) Cell growth, proliferation, colony formation, and invasion abilities were determined by CCK8, EdU, colony formation, and transwell assays in CAL‐27‐sh‐ALDH1L1 cells added LY294002 and SCC‐25‐LV‐ALDH1L1 cells added 740 Y‐P. Scale bar: 200 μm. These data represent at least three independent experiments. Error bars show mean ± SD. *p < 0.05, **p < 0.01, ***p < 0.001.

In order to confirm whether the anti‐proliferation effects of ALDH1L1 in OSCC cells are through the PI3K/Akt/Rb pathway, we added PI3K inhibitor LY294002 (10 μM, 1 h) to CAL‐27‐sh‐ALDH1L1 cells and PI3K activator 740 Y‐P (25 μg/mL, 2 h) to SCC‐25‐LV‐ALDH1L1 cells (Figure 6A, B). As shown in Figure 6C, D, LY294002 significantly reduced the growth and proliferation of ALDH1L1‐knockdown CAL‐27 cells, relative to control groups. Contrasting findings were established in ALDH1L1‐overexpression SCC‐25 cells with 740 Y‐P. In addition, the clonogenic ability and invasion of ALDH1L1‐knockdown CAL‐27 cells were significantly suppressed by LY294002. In contrast, 740 Y‐P significantly enhanced the clonogenic ability and invasion of ALDH1L1‐overexpression SCC‐25 cells (Figure 6 E, F). Therefore, inactivation of PI3K/Akt/Rb signaling plays a vital role in facilitating ALDH1L1‐mediated suppression of OSCC cell progression (Figure S6).

## 4 DISCUSSION

The best illustration of ALDH1L1 regulation is its silence in malignant tumors.^13^ The expression of ALDH1L1 at both mRNA and protein levels is usually absent in malignant tumors.^9^ Consistent with this finding, while immortalized non‐cancer cells express ALDH1L1, its protein levels were found to be undetectable in various cancer cell lines.^9^, ^22^ We found that expression levels of ALDH1L1 in OSCC tissues as well as cell lines were markedly suppressed when compared with those of ANTs and normal oral epithelial cells. The low levels could be a marker for aggressive tumor phenotypes. Therefore, suppressed ALDH1L1 expression is correlated with poor prognosis in hepatocellular carcinoma, sporadic pilocytic astrocytoma, and neuroblastoma.^23^, ^24^, ^25^ Here, we provided evidence that low ALDH1L1 expression in OSCC is associated with more malignant tumor phenotypes and poor survival. These findings suggested the potential value of ALDH1L1 as a prognostic biomarker for OSCC patients.

Different from other folate‐metabolizing enzymes, ALDH1L1 belongs to the aldehyde dehydrogenases family.^26^ In humans, this family has 19 genes that play roles in conversion of various aldehyde substrates into their equivalent acids.^27^ Each subunit of ALDH1L1 has three domains with different structures and functions. The amino‐terminal folate‐binding hydrolase domain resembles methionine‐tRNA formyltransferase and plays a major role, while the carboxyl‐terminal core domain is a structural and functional homolog of aldehyde dehydrogenases.^28^, ^29^, ^30^ The intermediate domain is a functional and structural homolog of the acyl carrier protein, while the two catalytic domains can communicate through the intermediate domain.^31^ Thus, ALDH1L1 is not a typical aldehyde dehydrogenase capable of oxidizing short‐chain aldehydes to their equivalent acids in vitro. However, it remains unknown whether this enzyme is a catalyst for various aldehyde dehydrogenase reactions.^32^ In fact, ALDH1L1 participates in a variety of biological pathways related to cellular proliferation in tumor cells. This enzyme competes with the same substrate, 10‐formyltetrahydrofolic acid, in the de novo purine synthesis reaction required for rapid tumor cell proliferation.^33^ In this regard, ALDH1L1 acts similar to anti‐FA drug against cancer progression. Natalia V reported that transient expression of ALDH1L1 suppresses cancer cell proliferation, resulting in cell death.^9^ Furthermore, ALDH1L1‐expressing A549 cells exhibited cell accumulation in the G0‐G1 phase and a decrease in cell numbers in the S phase.^16^ In our research, we confirmed that ALDH1L1 knockdown limits the occurrence of NADP^+^‐dependent 10‐formyltetrahydrofolic acid metabolic reactions and promotes rapid progression of OSCC cells both in vitro and in vivo. However, ALDH1L1 overexpression had the opposite results. The above data indicated that ALDH1L1 as the target of anti‐FA therapy could effectively inhibit OSCC growth. In addition, OSCC patients with low ALDH1L1 expression prudently take FA supplements in excess of the recommended dose during their illness. Of course, various of in vivo and clinical trials are needed to support this hypothesis.

Previously studies have shown that the effects of ALDH1L1 on cancer cell biological behavior mainly based on activation of JNK/p53, c‐Jun/Bid or p53/p21 pathways, but this is tumor type specific. Oleinik revealed that ALDH1L1 induces JNK1‐mediated phosphorylation of JNK2, and then p53 is directly phosphorylated by JNK2 at Ser6 in non‐small cell lung carcinoma.^34^ In addition, ALDH1L1 expression initiated the phosphorylation of JNK target c‐Jun and pro‐apoptotic protein, Bid, in p53‐deficient prostate cancer cell line, PC‐3.^35^ We used stable ALDH1L1‐overexpressing and ‐knockdown cell lines to investigate the key regulatory proteins of these classic pathways, it was found that the tumor suppressor effect of ALDH1L1 in OSCC may be through other pathways. In previous cell phenotypic experiments, we show that ALDH1L1 can promote the arrest of tumor cells in G0/G1 and G2/M phases. Therefore, we tested the classical signaling related to the G0/G1 and G2/M phases, and found that ALDH1L1 inhibits the activation of the PI3K/Akt/Rb pathway. By adding PI3K agonist and inhibitor, we confirmed that ALDH1L1‐induced inhibition of OSCC cell proliferative properties is through the deactivation of PI3K/Akt/Rb signaling.

Although there are still some deficiencies in this study, for example, we still do not know the reasons of ALDH1L1 downregulation in OSCC and the specific molecular mechanism of ALDH1L1 regulation of PI3K pathway, these problems will be the main direction to be solved in our follow‐up study. In summary, our data provide important insights into the role and underlying mechanisms of ALDH1L1 abnormal regulation in OSCC. We demonstrated that ALDH1L1 suppresses tumor proliferation, survival, cell cycle progression, and invasion via inhibiting PI3K/Akt/Rb signaling pathway. In addition, ALDH1L1 expression was inversely correlated with clinical T stage, pathological grade, and Ki‐67 index, and positively correlated with better survival in OSCC tissues. Combining the results of these studies, we propose that ALDH1L1 can be used as a prognostic marker and an important target for anti‐FA therapy in OSCC.

## AUTHORS CONTRIBUTION

Lizheng Qin and Zhengxue Han: Conceptualization, Methodology, Supervision, Project administration, Resources. Yi Qu and Ying He: Investigation, Data curation, Writing‐Original draft, Validation. Hanjin Ruan: Writing‐Review & Editing, Visualization.

## CONFLICT OF INTERESTS

The authors declare no conflict of interest.

## Supporting information


Figure S1
Figure S2
Figure S3
Figure S4
Figure S5
Figure S6

Table S1
Table S2
Table S3

## Data Availability

The data that support the findings of this study are available from the corresponding author upon reasonable request.

## References

[1] Economopoulou P , Kotsantis I , Kyrodimos E , Lianidou ES , Psyrri A . Liquid biopsy: An emerging prognostic and predictive tool in head and neck squamous cell carcinoma (HNSCC). Focus on circulating tumor cells (CTCs). Oral Oncol. 2017;74:83‐89. 29103757 10.1016/j.oraloncology.2017.09.012

[2] Kita A , Kasamatsu A , Nakashima D , et al. Activin B regulates adhesion, invasiveness, and migratory activities in Oral cancer: a potential biomarker for metastasis. J Cancer. 2017;8(11):2033‐2041. 28819404 10.7150/jca.18714 PMC5559965

[3] Sarode GS , Sarode SC , Maniyar N , Anand R , Patil S . Oral cancer databases: a comprehensive review. J Oral Pathol Med: Official Publication of the International Association of Oral Pathologists and the American Academy of Oral Pathology. 2018;47(6):547‐556. 10.1111/jop.12667 29193424

[4] Chien HT , Cheng SD , Liao CT , Wang HM , Huang SF . Amplification of the EGFR and CCND1 are coordinated and play important roles in the progression of Oral squamous cell carcinomas. Cancer. 2019;11(6):760. 10.3390/cancers11060760 PMC6627096 31159251

[5] Field MS , Stover PJ . Safety of folic acid. Ann N Y Acad Sci. 2018;1414(1):59‐71. 29155442 10.1111/nyas.13499 PMC5849489

[6] Yang C , Zhang J , Liao M , et al. Folate‐mediated one‐carbon metabolism: a targeting strategy in cancer therapy. Drug Discov Today. 2021;26(3):817‐825. 33316375 10.1016/j.drudis.2020.12.006

[7] Krupenko SA , Horita DA . The role of single‐nucleotide polymorphisms in the function of candidate tumor suppressor ALDH1L1. Front Genet. 2019;10:1013. 31737034 10.3389/fgene.2019.01013 PMC6831610

[8] Horita DA , Krupenko SA . Modeling of interactions between functional domains of ALDH1L1. Chem Biol Interact. 2017;276:23‐30. 28414156 10.1016/j.cbi.2017.04.011 PMC6289516

[9] Krupenko SA , Oleinik NV . 10‐formyltetrahydrofolate dehydrogenase, one of the major folate enzymes, is down‐regulated in tumor tissues and possesses suppressor effects on cancer cells. Cell Growth Differ. 2002;13(5):227‐236. 12065246

[10] Brosnan ME , MacMillan L , Stevens JR , Brosnan JT . Division of labour: how does folate metabolism partition between one‐carbon metabolism and amino acid oxidation? Biochem J. 2015;472(2):135‐146. 26567272 10.1042/BJ20150837

[11] Anguera MC , Field MS , Perry C , et al. Regulation of folate‐mediated one‐carbon metabolism by 10‐formyltetrahydrofolate dehydrogenase. J Biol Chem. 2006;281(27):18335‐18342. 16627483 10.1074/jbc.M510623200

[12] Ducker GS , Rabinowitz JD . One‐carbon metabolism in health and disease. Cell Metab. 2017;25(1):27‐42. 27641100 10.1016/j.cmet.2016.08.009 PMC5353360

[13] Li M , Sun Q , Wang X . Transcriptional landscape of human cancers. Oncotarget. 2017;8(21):34534‐34551. 28427185 10.18632/oncotarget.15837 PMC5470989

[14] Subramanian A , Tamayo P , Mootha VK , et al. Gene set enrichment analysis: a knowledge‐based approach for interpreting genome‐wide expression profiles. Proc Natl Acad Sci U S A. 2005;102(43):15545‐15550. 16199517 10.1073/pnas.0506580102 PMC1239896

[15] Oleinik NV , Krupenko NI , Priest DG , Krupenko SA . Cancer cells activate p53 in response to 10‐formyltetrahydrofolate dehydrogenase expression. Biochem J. 2005;391:503‐511. 16014005 10.1042/BJ20050533 PMC1276951

[16] Oleinik NV , Krupenko SA . Ectopic expression of 10‐formyltetrahydrofolate dehydrogenase in A549 cells induces G1 cell cycle arrest and apoptosis. Molecular Cancer Research: MCR. 2003;1(8):577‐588. 12805405

[17] Qu Y , He Y , Yang Y , et al. ALDH3A1 acts as a prognostic biomarker and inhibits the epithelial mesenchymal transition of oral squamous cell carcinoma through IL‐6/STAT3 signaling pathway. J Cancer. 2020;11(9):2621‐2631. 32201532 10.7150/jca.40171 PMC7066020

[18] Liu X , Ma B , Malik AB , et al. Bidirectional regulation of neutrophil migration by mitogen‐activated protein kinases. Nat Immunol. 2012;13(5):457‐464. 22447027 10.1038/ni.2258 PMC3330201

[19] Li H , Yu L , Liu J , et al. miR‐320a functions as a suppressor for gliomas by targeting SND1 and β‐catenin, and predicts the prognosis of patients. Oncotarget. 2017;8(12):19723‐19737. 28160566 10.18632/oncotarget.14975 PMC5386717

[20] Wen Y , Hansen Chen L , Zhang MW , Zhang F , Yang D , et al. Glycyrrhetinic acid induces oxidative/nitrative stress and drives ferroptosis through activating NADPH oxidases and iNOS, and depriving glutathione in triple‐negative breast cancer cells. Free Radic Biol Med. 2021;173:41‐51. 34271106 10.1016/j.freeradbiomed.2021.07.019

[21] Reddy D , Kumavath R , Ghosh P , Barh D . Lanatoside C induces G2/M cell cycle arrest and suppresses cancer cell growth by attenuating MAPK, Wnt, JAK‐STAT, and PI3K/AKT/mTOR signaling pathways. Biomolecules. 2019;9(12):792. 31783627 10.3390/biom9120792 PMC6995510

[22] Khan QA , Pediaditakis P , Malakhau Y , et al. CHIP E3 ligase mediates proteasomal degradation of the proliferation regulatory protein ALDH1L1 during the transition of NIH3T3 fibroblasts from G0/G1 to S‐phase. PloS One. 2018;13(7):e0199699. 29979702 10.1371/journal.pone.0199699 PMC6034817

[23] Rodriguez FJ , Giannini C , Asmann YW , et al. Gene expression profiling of NF‐1‐associated and sporadic pilocytic astrocytoma identifies aldehyde dehydrogenase 1 family member L1 (ALDH1L1) as an underexpressed candidate biomarker in aggressive subtypes. J Neuropathol Exp Neurol. 2008;67(12):1194‐1204. 19018242 10.1097/NEN.0b013e31818fbe1e PMC2730602

[24] Chen XQ , He JR , Wang HY . Decreased expression of ALDH1L1 is associated with a poor prognosis in hepatocellular carcinoma. Medical Oncology (Northwood, London, England). 2012;29(3):1843‐1849. 21987076 10.1007/s12032-011-0075-x

[25] Hartomo TB , Van Huyen PT , Yamamoto N , et al. Involvement of aldehyde dehydrogenase 1A2 in the regulation of cancer stem cell properties in neuroblastoma. Int J Oncol. 2015;46(3):1089‐1098. 25524880 10.3892/ijo.2014.2801

[26] Krupenko SA . FDH: an aldehyde dehydrogenase fusion enzyme in folate metabolism. Chem Biol Interact. 2009;178(1–3):84‐93. 18848533 10.1016/j.cbi.2008.09.007 PMC2664990

[27] Krupenko NI , Holmes RS , Tsybovsky Y , Krupenko SA . Aldehyde dehydrogenase homologous folate enzymes: evolutionary switch between cytoplasmic and mitochondrial localization. Chem Biol Interact. 2015;234:12‐17. 25549576 10.1016/j.cbi.2014.12.022 PMC4414694

[28] Tsybovsky Y , Donato H , Krupenko NI , Davies C , Krupenko SA . Crystal structures of the carboxyl terminal domain of rat 10‐formyltetrahydrofolate dehydrogenase: implications for the catalytic mechanism of aldehyde dehydrogenases. Biochemistry. 2007;46(11):2917‐2929. 17302434 10.1021/bi0619573

[29] Chumanevich AA , Krupenko SA , Davies C . The crystal structure of the hydrolase domain of 10‐formyltetrahydrofolate dehydrogenase: mechanism of hydrolysis and its interplay with the dehydrogenase domain. J Biol Chem. 2004;279(14):14355‐14364. 14729668 10.1074/jbc.M313934200

[30] Donato H , Krupenko NI , Tsybovsky Y , Krupenko SA . 10‐formyltetrahydrofolate dehydrogenase requires a 4′‐phosphopantetheine prosthetic group for catalysis. J Biol Chem. 2007;282(47):34159‐34166. 17884809 10.1074/jbc.M707627200

[31] Goldman ID , Chattopadhyay S , Zhao R , Moran R . The antifolates: evolution, new agents in the clinic, and how targeting delivery via specific membrane transporters is driving the development of a next generation of folate analogs. Curr Opin Investig Drugs. 2010;11(12):1409‐1423. 21154123

[32] Cook RJ , Lloyd RS , Wagner C . Isolation and characterization of cDNA clones for rat liver 10‐formyltetrahydrofolate dehydrogenase. J Biol Chem. 1991;266(8):4965‐4973. 1848231

[33] Krupenko SA , Wagner C , Cook RJ . Expression, purification, and properties of the aldehyde dehydrogenase homologous carboxyl‐terminal domain of rat 10‐formyltetrahydrofolate dehydrogenase. J Biol Chem. 1997;272(15):10266‐10272. 9092577 10.1074/jbc.272.15.10266

[34] Oleinik NV , Krupenko NI , Krupenko SA . Cooperation between JNK1 and JNK2 in activation of p53 apoptotic pathway. Oncogene. 2007;26(51):7222‐7230. 17525747 10.1038/sj.onc.1210526

[35] Ghose S , Oleinik NV , Krupenko NI , Krupenko SA . 10‐formyltetrahydrofolate dehydrogenase‐induced c‐Jun‐NH2‐kinase pathways diverge at the c‐Jun‐NH2‐kinase substrate level in cells with different p53 status. Mol Cancer Res. 2009;7(1):99‐107. 19147541 10.1158/1541-7786.MCR-08-0309 PMC2632845

