# Supplementary material for: Abnormal downregulation of 10‐formyltetrahydrofolate dehydrogenase promotes the progression of oral squamous cell carcinoma by activating PI3K/Akt/Rb pathway
Source: Cancer Med. 2022 Nov 6;12(5):5781–97. doi: 10.1002/cam4.5327 (PMC10028165; doi:10.1002/cam4.5327)
Supplement: Supplementary file 1 — Figure S1 Figure S2 Figure S3 Figure S4 Figure S5 Figure S6 [file CAM4-12-5781-s001.docx]

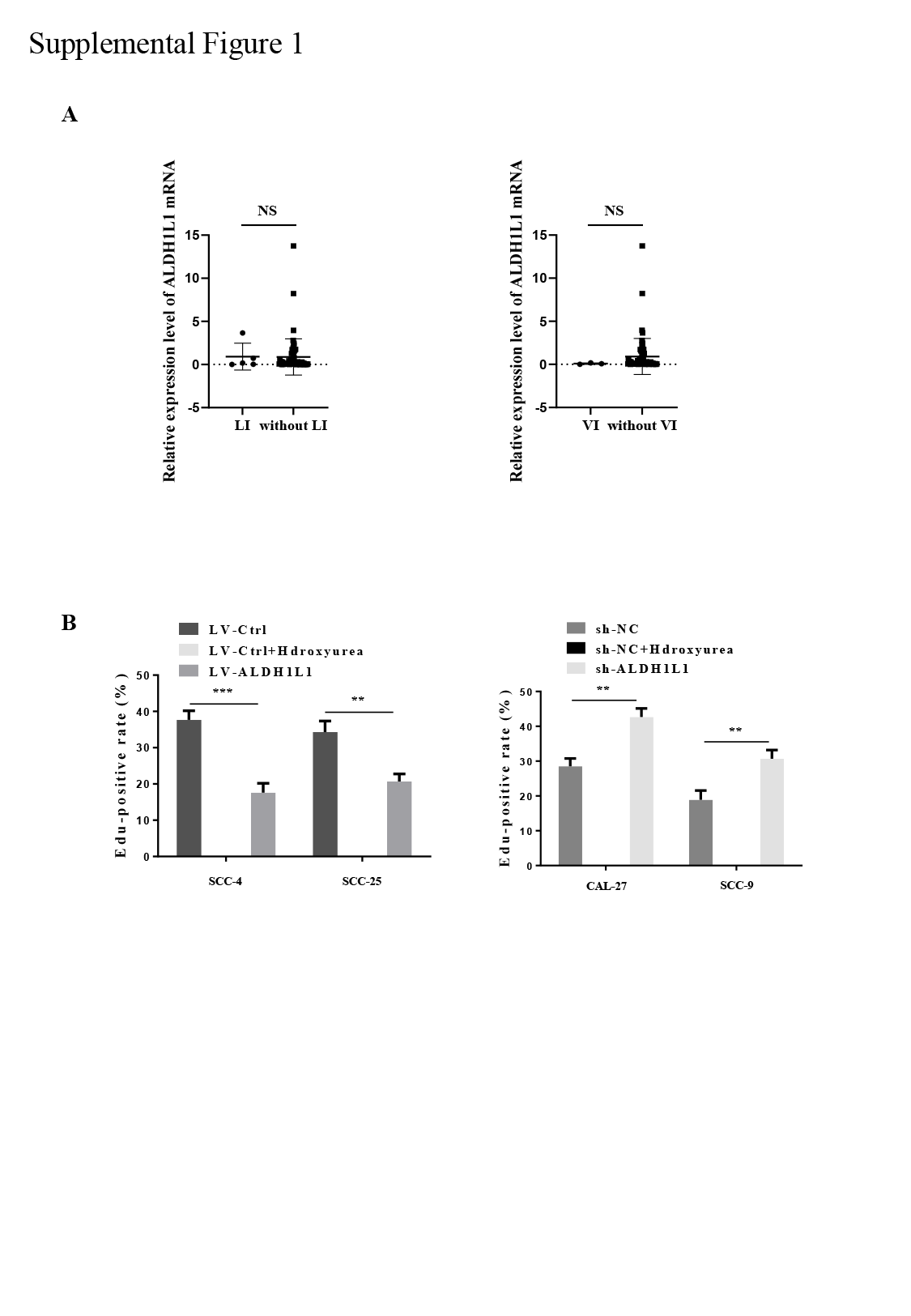


Supplemental Figure 1. (A) The correlation between ALDH1L1 mRNA expression levels and Lymphatic invasion (LI) or vascular invasion (VI) in OSCC patients. (B) The comparison of EdU-positive rates among the indicated cells. **p<0.01, ***p<0.001.


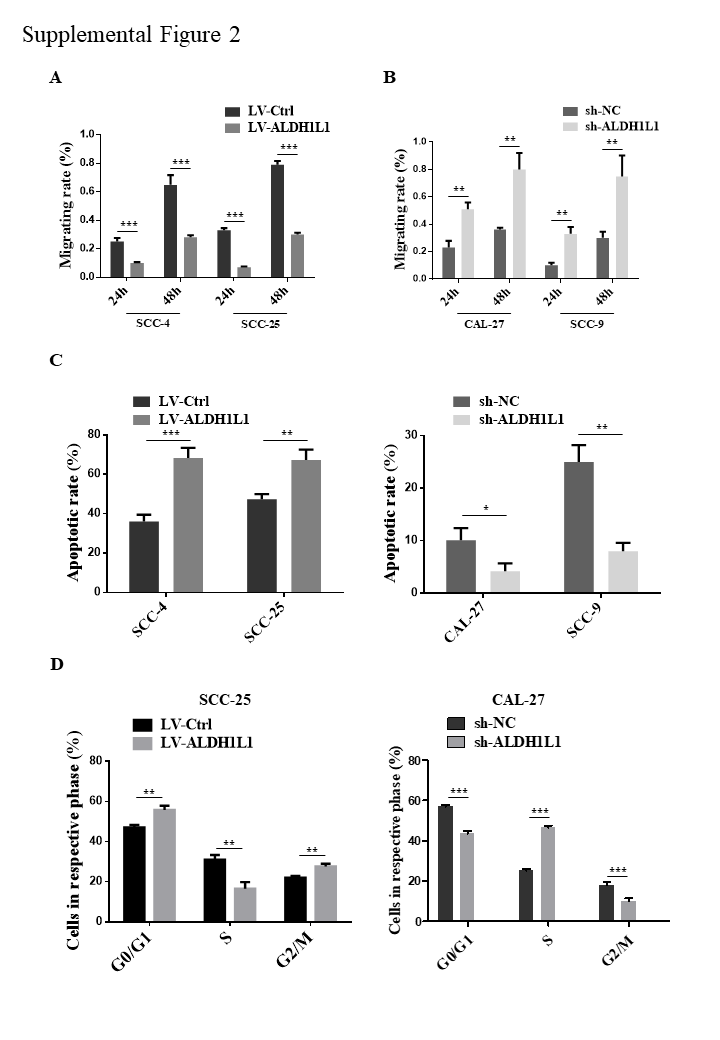


Supplemental Figure 2. (A) Up-regulation of ALDH1L1 inhibited the migration of SCC-4 and SCC-25 cells at 24h and 48h in wound-healing assays. (B) Down-regulation of ALDH1L1 enhanced the migration of CAL-27 and SCC-9 cells at 24h and 48h in wound-healing assays. (C) Flow cytometry analysis showed that the apoptotic rate of LV-ALDH1L1 SCC-4 and SCC-25 cells were significantly increased, while that of sh-ALDH1L1 CAL-27 and SCC-9 cells were significantly decreased. (D) Graphs represent the percentages of SCC-25 and CAL-27 cells in G0/G1, S, and G2/M phases of the cell cycle. *p<0.05, **p<0.01, ***p<0.001.


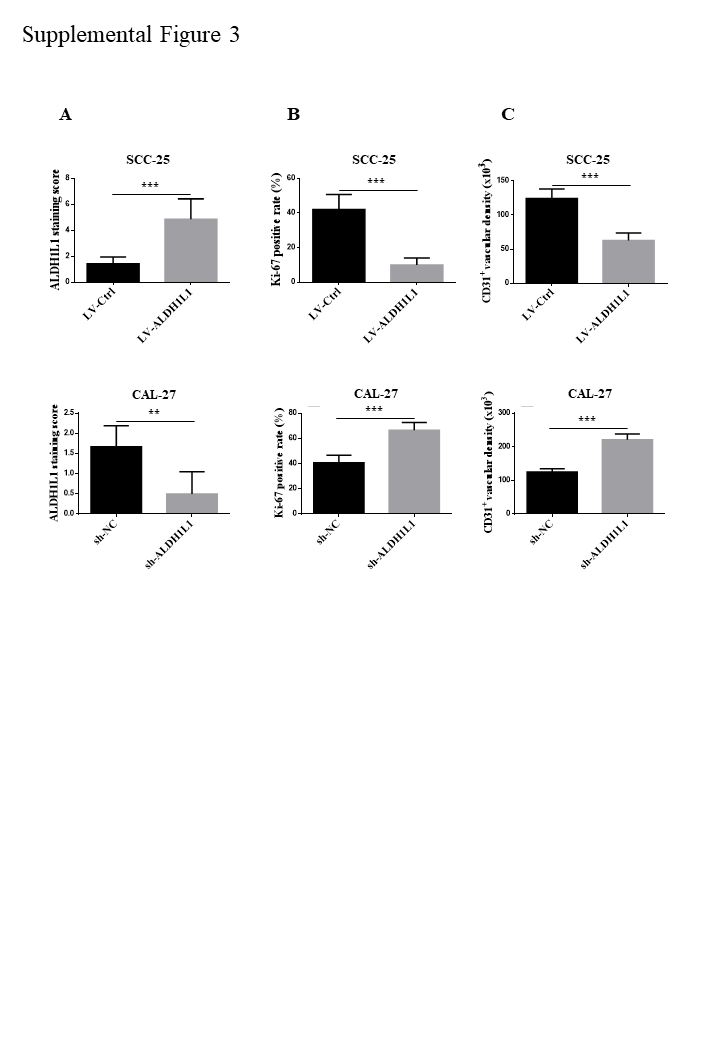


Supplemental Figure 3. (A) The ALDH1L1 staining scores in tumor tissues of the LV-ALDH1L1 and sh-ALDH1L1 groups were compared with the corresponding control groups. (B) Results of Ki-67 staining in mice transplanted with LV-ALDH1L1 and sh-ALDH1L1 cells and their corresponding control cells were evaluated by the positive cell rate. (C) Vascular density was quantified by measuring the vessel as (per 200× field, 5 fields per section) using the Image J software, based on CD31 expression. Results are mean ± SD of three independent experiments and analyzed by Student’s t-test. *p<0.05, **p<0.01, ***p<0.001.


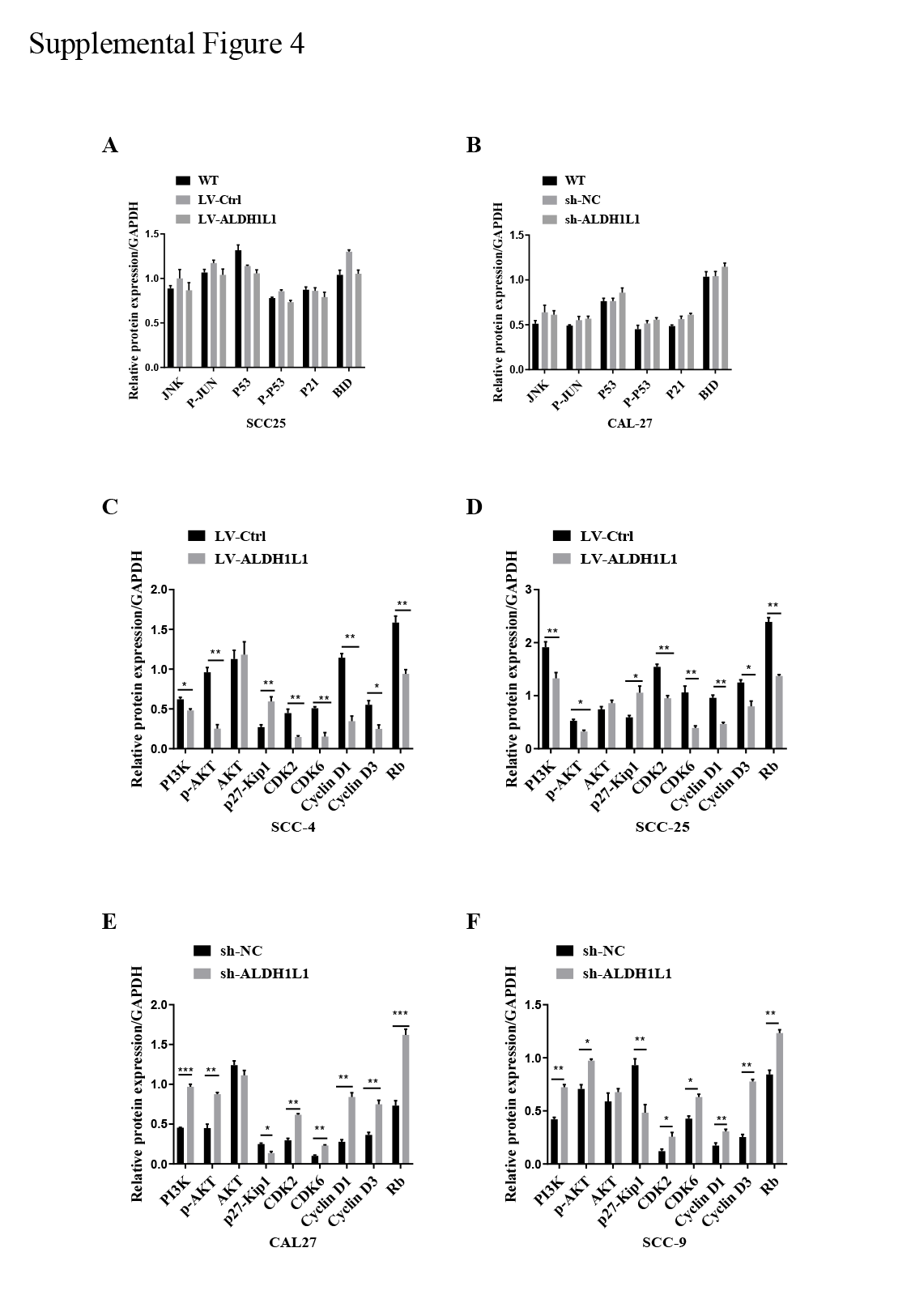


Supplemental Figure 4. Densitometric analyses of western blots on Figure 5C (A and B) and D (C-F). *p<0.05, **p<0.01, ***p<0.001.


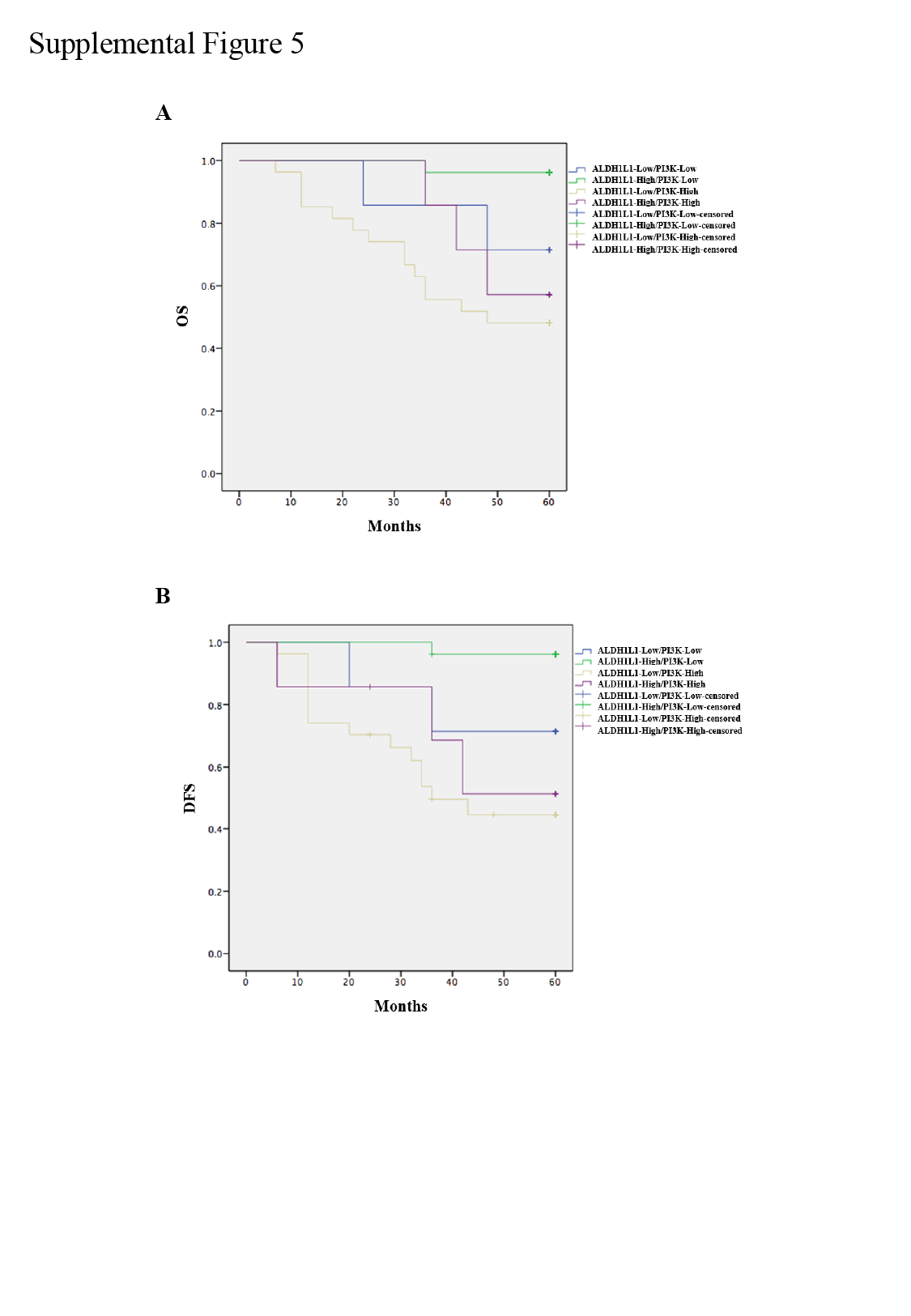


Supplemental Figure 5. Kaplan-Meier survival analysis was performed to estimate the relationship between different ALDH1L1/PI3K expression combinations in tumor tissues and the overall survival (OS) (A) and disease-free survival (DFS) (B) of OSCC patients.


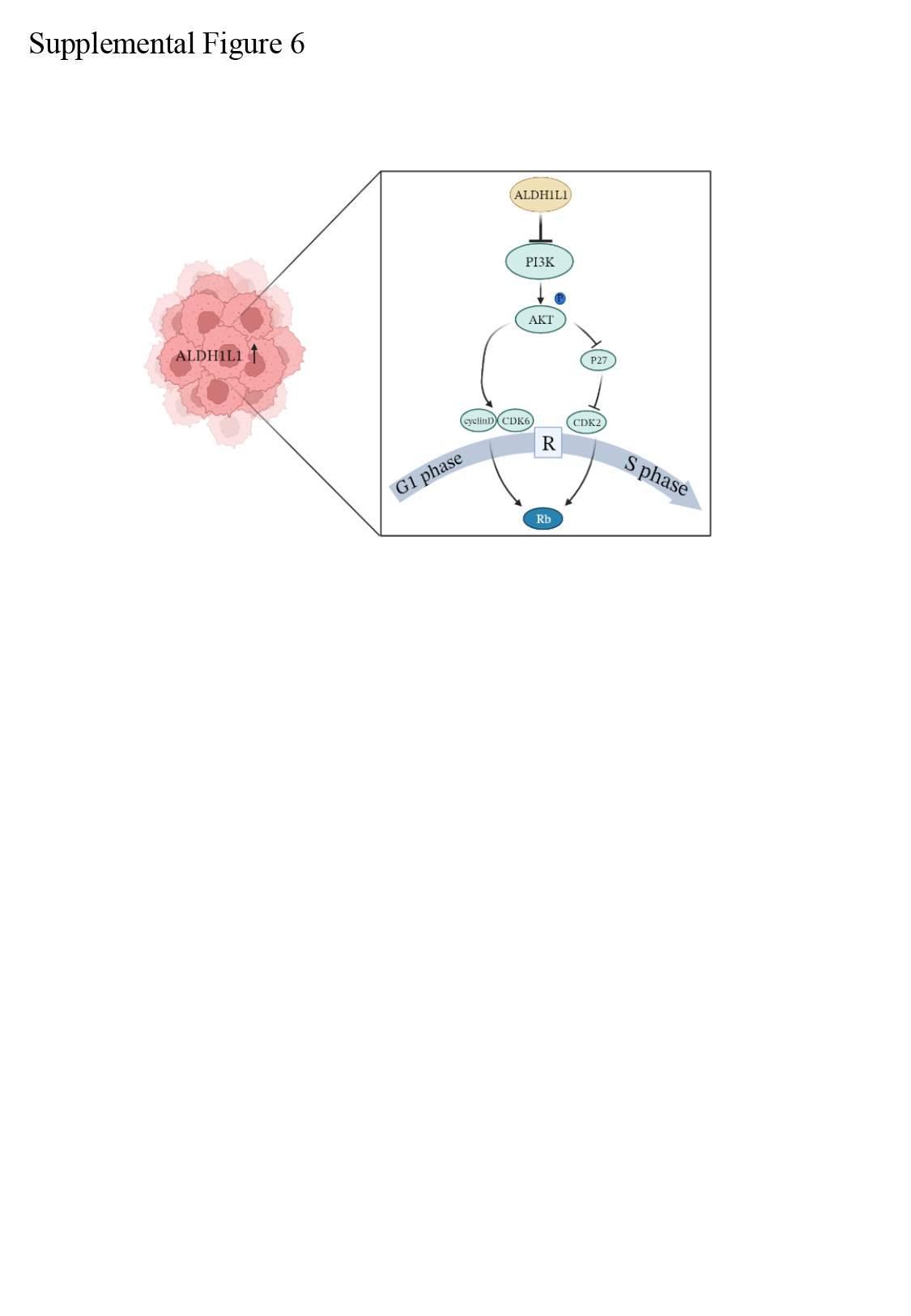


Supplemental Figure 6. ALDH1L1 regulates OSCC progression via the PI3K/Akt/Rb pathway.
