# Supplementary material for: Abnormal downregulation of 10‐formyltetrahydrofolate dehydrogenase promotes the progression of oral squamous cell carcinoma by activating PI3K/Akt/Rb pathway
Source: Cancer Med. 2022 Nov 6;12(5):5781–97. doi: 10.1002/cam4.5327 (PMC10028165; doi:10.1002/cam4.5327)
Supplement: Supplementary file 2 — Table S1 Table S2 Table S3 [file CAM4-12-5781-s002.docx]

Supplemental Tables

| **Supplemental Table 1. Sequences of qRT-PCR primers** | |
| --- | --- |
| **Name** | **Sequences** |
| **ALDH1L1-Forward** | **TCCAGACCTTCCGCTACTTTG** |
| **ALDH1L1-Reverse** | **CAGGGGATAGTTCCAGGGGAT** |
| **GAPDH-Forward** | **CTGGGCTACACTGAGCACC** |
| **GAPDH-Reverse** | **AAGTGGTCGTTGAGGGCAATG** |
| **JNK-Forward** | **TCTGGTATGATCCTTCTGAAGCA** |
| **JNK-Reverse** | **TCCTCCAAGTCCATAACTTCCTT** |
| **p53-Forward** | **GAGGTTGGCTCTGACTGTACC** |
| **p53-Reverse** | **TCCGTCCCAGTAGATTACCAC** |
| **p21-Forward** | **CGATGGAACTTCGACTTTGTCA** |
| **p21-Reverse** | **GCACAAGGGTACAAGACAGTG** |
| **Bid-Forward** | **ATGGACCGTAGCATCCCTCC** |
| **Bid-Reverse** | **GTAGGTGCGTAGGTTCTGGT** |
| **FOXM1-Forward** | **GGAGCAGCGACAGGTTAAGG** |
| **FOXM1-Reverse** | **GTTGATGGCGAATTGTATCATGG** |

| **Supplemental Table 2. Antibodies for Western blot and Immunohistochemistry** | | |
| --- | --- | --- |
| **Name** | **Catlog No.** | **Company** |
| **Rabbit anti-human ALDH1L1** | **ab175198** | **Abcam** |
| **Mouse anti-human GAPDH** | **97166** | **Abclonal** |
| **Rabbit anti-human JNK1/2/3** | **ab179461** | **Abcam** |
| **Rabbit anti-human p-JNK1/2/3** | **ab124956** | **Abcam** |
| **Rabbit anti-human p53** | **2527** | **Cell Signaling Technology** |
| **Rabbit anti-human p-p53** | **82530** | **Cell Signaling Technology** |
| **Rabbit anti-human p21** | **2947** | **Cell Signaling Technology** |
| **Mouse anti-human Bid** | **8762** | **Cell Signaling Technology** |
| **Rabbit anti-human PI3K** | **4257** | **Cell Signaling Technology** |
| **Rabbit anti-human p-PI3K** | **17366** | **Cell Signaling Technology** |
| **Rabbit anti-human Akt** | **4691** | **Cell Signaling Technology** |
| **Rabbit anti-human p-Akt** | **4060** | **Cell Signaling Technology** |
| **Mouse anti-human p27 Kip1** | **3698** | **Cell Signaling Technology** |
| **Rabbit anti-human CDK2** | **18048** | **Cell Signaling Technology** |
| **Mouse anti-human CDK6** | **3136** | **Cell Signaling Technology** |
| **Rabbit anti-human Cyclin D1** | **55506** | **Cell Signaling Technology** |
| **Mouse anti-human Cyclin D3** | **2936** | **Cell Signaling Technology** |
| **Rabbit anti-human Rb** | **ab181616** | **Abcam** |
| **Rabbit anti-human Ki-67** | **ab15580** | **Abcam** |
| **Mouse anti-human CD31** | **3528** | **Cell Signaling Technology** |

| **Supplemental Table 3. Target Sequences of shRNAs** | |
| --- | --- |
| **Name** | **Target Sequences** |
| **sh-ALDH1L1** | **ctCATCCTCTTTGGGAATGAT** |
| **sh-NC** | **TTCTCCGAACGTGTCACGT** |

| **Supplemental Table 4. Sequences of cloning primers** | |
| --- | --- |
| **Name** | **Sequences** |
| **LV-ALDH1L1-Forward** | **GAGGATCCCCGGGTACCGGTCGCCACCATGAAGATTGCAGTGATTGGAC** |
| **LV-ALDH1L1-Reverse** | **CACACATTCCACAGGCTAGCTCAGGTTGCGGTTGGGTCTGGC** |
